# Brown-Séquard Syndrome Following a Thoracic Spine Stab Wound: A Case Report

**DOI:** 10.7759/cureus.46917

**Published:** 2023-10-12

**Authors:** Tiago S Moreira, Susana Moreira, Ana Teixeira-Vaz

**Affiliations:** 1 Physical Medicine and Rehabilitation Department, Centro Hospitalar Universitário de São João, Porto, PRT

**Keywords:** brown-séquard syndrome, physical medicine and rehabilitation, trauma, knife, penetrating spinal cord injury, spinal cord injury

## Abstract

Nonmissile penetrating spine injury represents a small percentage of spinal cord injuries (SCIs), estimated at 0.8% in Western countries. This paper presents a detailed case report of a 28-year-old man with a history of substance use who suffered multiple injuries following a violent incident. The patient was found with a knife embedded in his thoracic spine, prompting immediate medical intervention. Computed tomography and postoperative magnetic resonance imaging revealed the extent of spinal cord and anatomical involvement. A thorough physical medicine and rehabilitation evaluation was conducted post-surgery, leading to a diagnosis of Brown-Séquard syndrome with associated sensorimotor deficits. This paper highlights the challenges posed by penetrating SCIs while reviewing the literature.

## Introduction

Nonmissile penetrating spinal injuries (NMPSIs) constitute a relatively rare occurrence within the spectrum of spinal cord injuries (SCIs), accounting for approximately 0.8% of cases in Western nations. However, in regions characterized by elevated levels of violence, this incidence can surge dramatically, reaching as high as 26% [1]. Within this subset, we encounter spinal stab wounds, frequently stemming from acts of assault, imparting a discernible, pointed trajectory of harm. These injuries mainly affect the lower cervical and upper thoracic region and encompass both immediate repercussions arising from the direct trauma inflicted by the penetrating object, as well as delayed consequences, encompassing the specters of infection, spinal fluid leakage, and enduring chronic pain [2].

Brown-Séquard syndrome (BSS) is a rare form of incomplete SCI consisting of ipsilateral upper motor neuron paralysis and loss of proprioception with contralateral pain and temperature sensation deficits resulting from hemisection or lateral injury to the spinal cord. It is the most common neurological pattern due to a transverse hemisection of the spinal cord [3]. The role of surgery in the treatment of penetrating spinal injury has been controversial in the literature [4,5].

Here, we present the case of a 28-year-old man who sustained a stab injury during a severe altercation, leaving the patient with a T8 BSS. We discuss the intricacies of the management of such cases, along with a review of the literature.

## Case presentation

**Figure 1:**
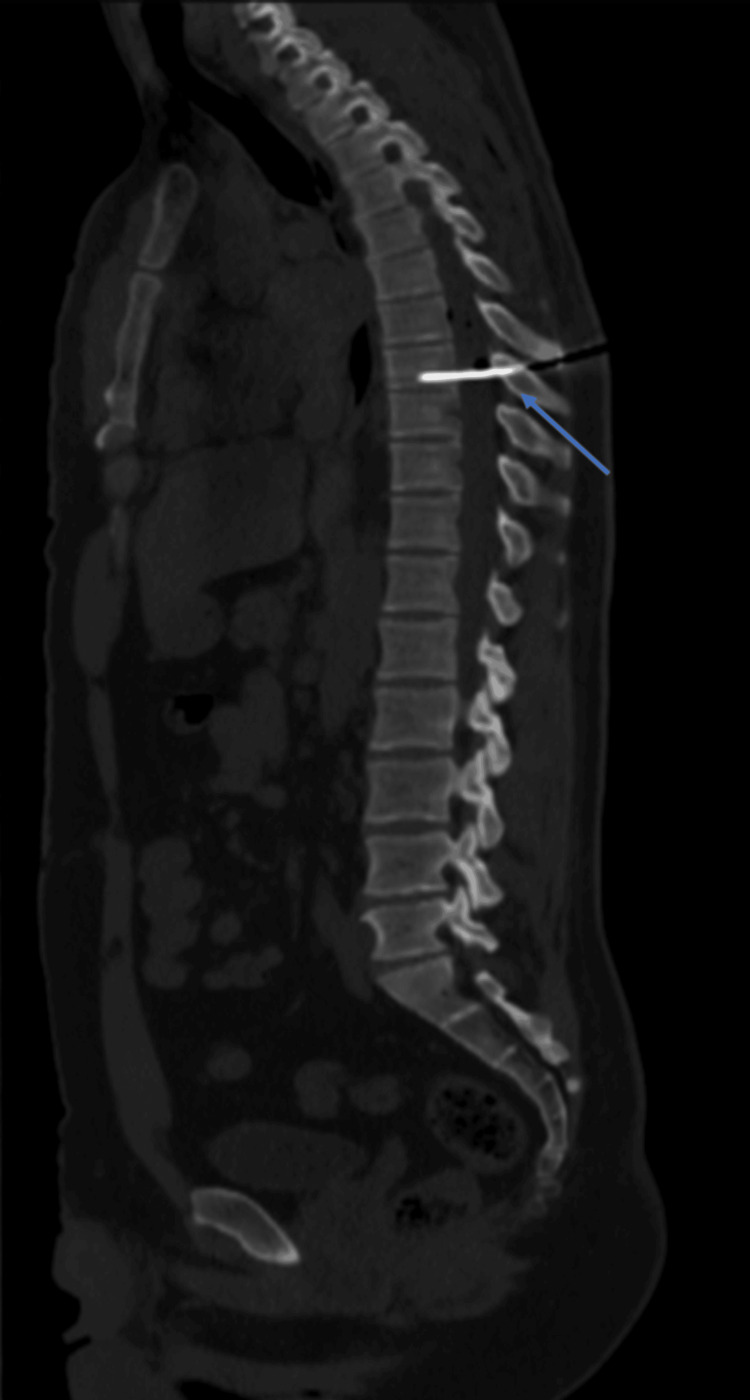
A computed tomography scan depicting the penetrating object crossing the T8 vertebral body (blue arrow).

**Figure 2:**
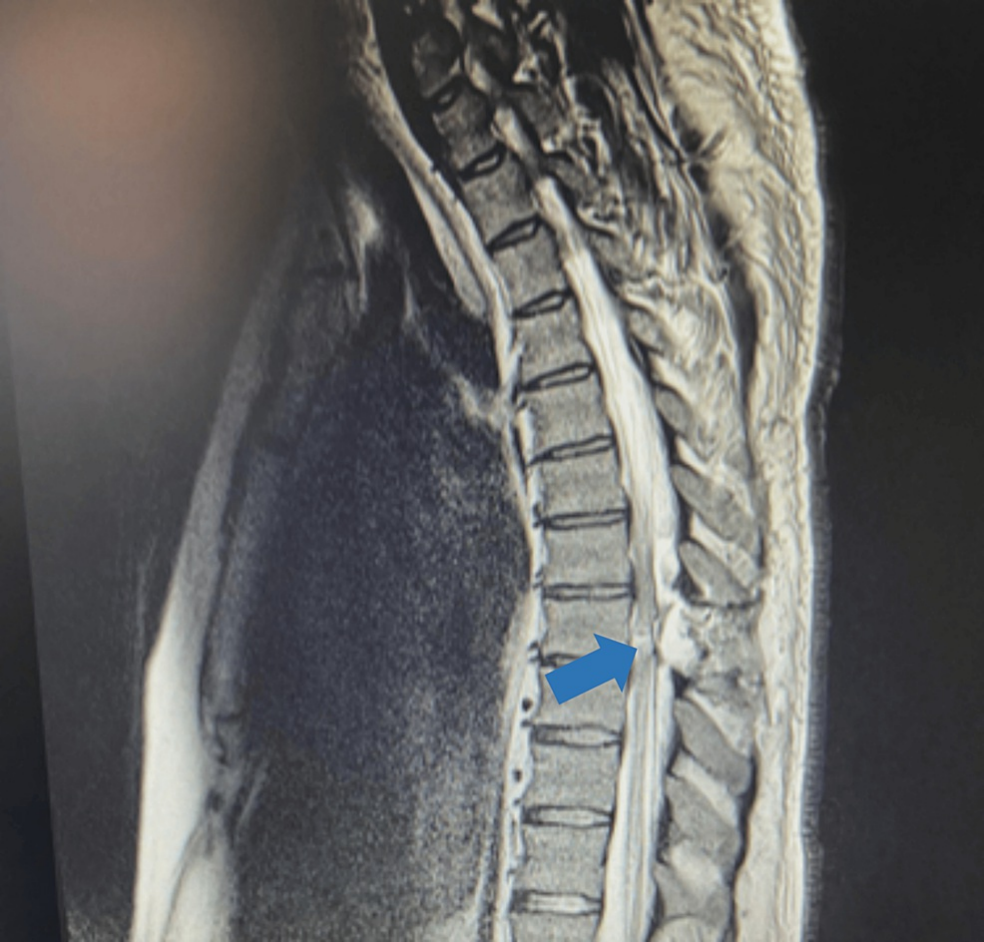
T2-weighted magnetic resonance imaging revealing an altered signal area in the right hemimedulla (blue arrow).

A 28-year-old man with a history of regular drug abuse was admitted to our emergency department due to multiple injuries sustained during a severe altercation. Upon admission, the patient was found conscious in a prone position with a knife protruding from his thoracic spine. He was tachycardic but otherwise vitally stable. Computed tomography (CT) imaging delineated the trajectory of the knife, illustrating its penetration through the interlaminar space, middle spinal cord parenchyma, and into the vertebral body at the T8 level, traversing the spinal canal (Figure 1). Given the patient’s stable condition and the absence of pulmonary or vascular complications, surgical intervention was deemed appropriate. The patient underwent an emergent neurosurgical procedure to extract the bladed weapon. Subsequent postoperative magnetic resonance imaging (MRI) revealed an altered signal area in the right hemimedulla, extending contralaterally (Figure 2). A Physical Medicine and Rehabilitation (PMR) evaluation was conducted on the fifth day post-surgery. At that time, the patient was awake and calm and exhibited preserved upper limb muscle strength (as per the Medical Research Council scale), except for the left wrist extensors (0/5). In the lower limbs, muscle strength was maintained on the left side, whereas a significant decrease was observed on the right side, affecting the hip flexors (1/5), knee extensors (3/5), foot and ankle dorsiflexors, plantar flexors (0/5), and hallux extensors (0/5). Additionally, tactile hypoesthesia was noted from T9 to S4/5 on the left side and from T9 to T12 on the right side, along with thermoalgic hypoesthesia at C6 and from T8 to S4/5 on the left side and T9 to T12 on the right side. Proprioception loss was observed on the right side. Sphincter control, however, remained intact. Based on these observations, a diagnosis of BSS with paraplegia AIS C NLI T8 (American Spinal Injury Association Impairment Scale C), along with a sensorimotor lesion of the radial nerve, was hypothesized. In this clinical context, an electromyography evaluation was requisitioned, unveiling outcomes consistent with severe axonal injury, predominantly affecting the sensory and motor fibers of the left radial nerve. In response to these findings, an intensive and tailored PMR program was promptly initiated during the patient’s hospitalization. Concurrently, a surgical neurorrhaphy procedure for the radial nerve was performed by the Plastic Surgery team. The patient was then transferred to an inpatient rehabilitation service to continue the rehabilitation program and received specialized care over one month. During this time, the patient received daily physical therapy sessions in the morning, which included a combination of range of motion exercises, strength training, balance and coordination exercises, and gait training. Additionally, the patient underwent occupational therapy to improve self-care skills, upper limb strength, and adapt to assistive devices. Sensory re-education techniques were employed to enhance the perception of touch, temperature, and proprioception. After the 30-day inpatient rehabilitation program, the patient demonstrated substantial progress. Notably, the patient achieved independence in transfers and exhibited a robust palmar grip in the left hand. Moreover, the patient successfully attained knee flexion in the right leg against the force of gravity. He was then transferred to another rehabilitation facility.

## Discussion

The management of BSS, particularly concerning surgical intervention, remains a subject of debate, particularly in cases of penetrating injuries where surgery has not demonstrated clear benefits over conservative approaches. Surgical intervention is typically recommended in specific situations, such as the presence of a foreign body, cerebrospinal fluid leakage, persistent compression of the spinal cord or nerve roots, sepsis, or progressive neurological deterioration.

Damage to the spinal cord as a type of incomplete SCI can develop into BSS, which often occurs in the cervical spinal cord region. Annually, the United States witnesses approximately 12,000 cases of SCIs. Among these cases, BSS is a relatively rare occurrence, representing 1% to 4% of all SCI cases [6].

Furthermore, SCIs resulting from stab injuries are exceptionally uncommon, accounting for less than 1.5% of the total reported cases.

The classic presentation of BSS is not a frequent occurrence. In cases where a knife blade penetrates through the interlaminar space, as in the scenario under consideration, the spinal cord sustains localized damage at the point of impact. Furthermore, countercoup damage emerges due to the spinal cord pushing against the opposite side of the spinal canal wall. It is worth noting that the severity of neurological damage and the level of neurological impairment can be influenced by the extent of edema within the spinal cord.

To pinpoint the precise relationship of the foreign object with anatomical structures, imaging studies are imperative, typically involving plain film X-rays or CT scans. MRI may not be a feasible option due to the physical characteristics of the penetrating object. However, MRI is considered the gold standard to rule out surgical causes of neurological deficits [7].

Interestingly, the extent of pathological changes in the spinal cord resulting from incomplete injuries caused by penetrating stab wounds may not consistently align with the anticipated recovery levels. Remarkably, in the majority of cases, functional recovery is notably favorable, with reports indicating significant improvement in 61% of stab wound injuries. This prognosis surpasses that of SCIs stemming from gunshot wounds and motor vehicle accidents [8].

The debate surrounding the management of penetrating SCIs continues to pit conservative and operative approaches against each other. Certain instances have presented promising results when surgical intervention is employed, utilizing techniques such as hemilaminotomy and exploration of the initial wound to remove foreign objects, followed by the crucial step of dural repair. In cases where the risk of infection is deemed low, any leaks of cerebrospinal fluid can be mended through sutures, and patients may commence a prophylactic antibiotic regimen [9]. Moreover, several studies suggest that prioritizing conservative management over aggressive surgical foreign body removal may be advisable to prevent the occurrence of new iatrogenic deficits, and therefore, indications for surgery must be clear [6,9]. In certain studies, it has been demonstrated that both surgical and conservative management approaches exhibit comparable effectiveness in addressing penetrating SCIs, regardless of the severity of the injury [10].

This case exemplifies a presentation of BSS resulting from a stab wound injury, exhibiting neurologic symptoms that align with the established findings in prior research.

In a substantial number of SCIs stemming from stab wounds, surgical intervention, often involving exploration, becomes a necessity. This surgical approach aims to address several critical aspects, including the removal of foreign objects and bone fragments that might exert pressure on neural elements, the confirmation and repair of cerebrospinal fluid leaks, and thorough irrigation of the injured area.

Nevertheless, it is worth noting that in cases of stab injuries where there are no discernible lesions exerting pressure on the neural components, opting for conservative treatment alone emerges as a rational and viable therapeutic strategy. This underscores the importance of individualized care tailored to the specific characteristics of each injury, with a preference for noninvasive approaches when suitable.

In this case, after the intensive 30-day inpatient rehabilitation program, the patient demonstrated significant progress. Notably, the patient achieved independence in transfers, a critical milestone in regaining functional autonomy. Moreover, the patient exhibited a robust palmar grip in the left hand, indicating substantial improvement in upper limb strength and dexterity. Another noteworthy achievement was the successful attainment of knee flexion in the right leg against the force of gravity, a key indicator of enhanced mobility.

## Conclusions

Penetrating SCIs pose intricate challenges in diagnosis, treatment, and rehabilitation. The treatment modality depends on the causes and the condition of the lesion. When considering surgical options, it is crucial to adhere to clear indications, and whenever feasible, prioritize conservative management over a more assertive surgical approach. This approach minimizes the risk of iatrogenic complications. Conservative treatment can be the preferred approach in BSS caused by a stab injury unless there is evidence of a lesion exerting pressure on the neural components. PMR programs play a pivotal role in maximizing the functionality, independence, and participation of these patients.
